# Brazilian undergraduate nursing students’ critical thinking need to be increased: a cross-sectional study

**DOI:** 10.1590/0034-7167-2022-0315

**Published:** 2022-11-28

**Authors:** Andréa Aparecida Gonçalves Nes, Fernando Riegel, Jussara Gue Martini, Jaroslav Zlamal, Paula Bresolin, Andrea Gomes da Costa Mohallem, Simen Alexander Steindal

**Affiliations:** I Lovisenberg Diaconal University College. Oslo, Norway; II Universidade Federal do Rio Grande do Sul. Rio Grande do Sul, Porto Alegre, Brazil; III Universidade Federal de Santa Catarina. Santa Catarina, Florianópolis, Brazil; IV Faculdade Israelita de Ciências da Saúde Albert Einstein. São Paulo, São Paulo, Brazil; V VID Specialized University. Oslo, Norway

**Keywords:** Critical Thinking, Cross Sectional Studies, Health Care, Nursing Education, Nursing Students, Pensamento Crítico, Estudos Transversais, Serviços de Saúde, Educação em Enfermagem, Estudantes de Enfermagem, Pensamiento Crítico, Estudios Transversales, Cuidado de la Salud, Educación en Enfermería, Estudiantes de Enfermería

## Abstract

**Objectives:**

to map Brazilian undergraduate nursing students’ critical thinking level and investigate the correlation between selected sociodemographic data and critical thinking domains.

**Methods:**

in this descriptive cross-sectional study, participants’ (N=89) critical thinking was assessed using the Health Science Reasoning Test. Correlation between critical thinking domains and sociodemographic data was assessed using the Pearson correlation coefficient.

**Results:**

the overall results showed a moderate level of participants’ critical thinking (mean = 70.7; standard deviation 5.7). A poor performance was identified in 5 of the 8 critical thinking domains. A significant positive correlation was found between education period and critical thinking (p<.001).

**Conclusions:**

poor level in students critical thinking domains may lead to negative consequences for their learning outcomes. Further studies should be carried out to confirm our results, in addition to investigation of teaching methods that encourage and ensure the development of students’ critical thinking skills during nursing education.

## RESUMO

**Objetivos:**

mapear o nível de pensamento crítico de estudantes brasileiros de graduação em enfermagem e investigar a correlação entre os dados sociodemográficos selecionados e os domínios do pensamento crítico.

**Métodos:**

neste estudo transversal descritivo, o pensamento crítico dos participantes (N=89) foi avaliado por meio do *Health Science Reasoning Test*. A correlação entre os domínios do pensamento crítico e os dados sociodemográficos foi avaliada pelo coeficiente de correlação de Pearson.

**Resultados:**

os resultados gerais mostraram um nível moderado de pensamento crítico dos participantes (média=70,7; desvio padrão 5,7). Foi identificado um desempenho ruim em 5 dos 8 domínios do pensamento crítico. Foi encontrada correlação positiva significativa entre escolaridade e o pensamento crítico (p<0,001).

**Conclusões:**

baixos níveis nos domínios de pensamento crítico em estudantes podem levar a consequências negativas para seus resultados de aprendizagem. Novos estudos devem ser realizados para confirmar nossos resultados, além da investigação de métodos de ensino que incentivem e garantam o desenvolvimento das habilidades de pensamento crítico dos estudantes durante a formação em enfermagem.

## RESUMEN

**Objetivos:**

mapear el nivel de pensamiento crítico de los estudiantes brasileños de pregrado en enfermería e investigar la correlación entre los datos sociodemográficos seleccionados y los dominios del pensamiento crítico.

**Métodos:**

en este estudio transversal descriptivo, el pensamiento crítico de los participantes (N=89) fue evaluada mediante el *Health Science Reasoning* Test. La correlación entre los dominios de pensamiento crítico y los datos sociodemográficos se evaluó mediante el coeficiente de correlación de Pearson.

**Resultados:**

los resultados generales mostraron un nivel moderado de pensamiento crítico de los participantes (media=70,7; desviación estándar 5,7). Se identificó un desempeño deficiente en 5 de los 8 dominios del pensamiento crítico. Se encontró una correlación positiva significativa entre educación y pensamiento crítico (p<0,001).

**Conclusiones:**

los bajos niveles de los dominios del pensamiento crítico en los estudiantes pueden tener consecuencias negativas para sus resultados de aprendizaje. Se deben realizar más estudios para confirmar nuestros resultados, además de la investigación de métodos de enseñanza que fomenten y aseguren el desarrollo de habilidades de pensamiento crítico de los estudiantes durante su formación en enfermería.

## INTRODUCTION

Critical thinking (CT) describes a conception of mental processes or a set of skills or attitudes^(1)^. The Delphi Report defines CT as the process of making a reflective judgement^(2)^ about what to believe or do in a given context^(3)^. It is a reflective process, as it demands self-monitoring and self-correction.

An essential element of this definition is that CT is exercised in a context and requires discipline-specific knowledge; the definition focuses on individuals’ inclination to engage in CT, especially critical judgement^(2)^. The inclination to engage in CT can be expressed as attitudes or habits that are integral to individuals’ actions and beliefs^(4)^.

CT skills should be fostered and measured in health care students at various levels of education^(5-6)^. In the United States of America (USA), CT skills have been integrated and assessed in preregistration programmes since 1989^(7)^. Several systematic reviews of CT skills in nursing and other health care professions emphasise the importance of CT in providing safe, effective care and recommend that nursing and other health care professional programmes focus on nurturing CT skills^(8-10)^.

Despite the importance of CT in the nursing profession, studies report a lack of CT in undergraduate nursing students (UNS)^(11-12)^. A study found that Iranian UNS’ CT skills did not significantly change during their nursing education^(12)^. A study that measured CT skills in UNS, at entry and exit (three years later), using the Health Sciences Reasoning Test (HSRT), indicated that UNS entry CT scores significantly correlated to academic performance and predicted UNS risk of course failure and ability to complete the nursing programme in three years^(13)^. Another study examined CT skills among UNS in Australia to obtain a profile and determine demographic predictors of CT using the HSRT. The results suggest that year of study predicted higher CT scores for all domains except the subscale analysis^(7)^. Furthermore, a randomized controlled trial compared the effectiveness of problem-based learning (PBL) associated with the active learning model versus conventional learning in improving CT among Brazilian UNS in a basic life support course. CT was evaluated using the California Critical Thinking Dispositions Inventory (CCTDI), and CT skills were evaluated using the California Critical Thinking Skills Test (CCTST). There were no significant variations between the pre- and post-tests for overall CCTDI and CCTST scales, but there was improvement in disposition analyticity and skill analysis in the experimental group^(14)^.

Since nurses are constantly managing complex situations and caring for patients at all hours, it is important to assess UNS’ CT levels at multiple stages of their nursing education so that nurse educators can tailor learning activities to enhance CT and ensure the desired outcome. To the best of our knowledge, no studies have used the HSRT to map Brazilian UNS’ CT skill levels.

## OBJECTIVES

To map Brazilian UNS’ CT skill levels using the HSRT and investigate and its correlation with chosen sociodemographic data.

Considering the importance of CT in the nursing profession, we posed the following research questions: what are the CT skill levels among UNS in Brazilian public and private universities? What are the correlations between UNS’ CT skills and age, sex, level of education, study period, time of testing performance and previous courses in CT? What recommendations to encourage CT in UNS emerge from the study’s results?

## METHODS

### Ethical aspects

This study was approved by the Research Ethics Committee of the *Universidade Federal de Santa Catarin*a (UFSC). The research followed the Resolution 466/2012 principles. Participation was voluntary, and participants were assured of information anonymity and confidentiality.

### Study design

The present study was an observational cross-sectional. The Equator instrument that was used to guide the study methodology was the STROBE checklist for cross-sectional studies.

### Sample

From May 2020 to May 2021, UNS were invited to participate in the study through announcements on the UFSC website and on the research team’s social networks. Due to the difficulty of recruiting participants, the invitation was extended and reproduced by several public and private universities in Brazil. Contact with other educational institutions was stablished through directed electronic correspondence with the deans of health sciences faculties.

### Inclusion and exclusion criteria

The study employed convenience sampling to recruit UNS (from all semesters of their programmes) from 28 public universities and 30 private universities in Brazil. Graduated nursing students such as residency, master, and doctoral students in any graduate specialisation were excluded. Students interested in participating in the study contacted the responsible researcher (FR) and subsequently received detailed information. Those who agreed to participate in the study signed the Informed Consent Form.

### Instrument applied for measuring critical thinking skills

CT skills were measured by the HSRT. Insight Assessment owns the copyright and licenses to use the HSRT around the world. The research group paid for this license to apply the HRST in this study. The HSRT has for over 12 years been one of the main instruments for objectively measuring CT; it comprises eight measures (domains) of CT skills: Analysis, Interpretation, Inference, Evaluation, Explanation, Deductive Reasoning, Inductive Reasoning, and Quantitative Reasoning (Numeracy), as described in Chart 2. The test features 38 multiple-choice questions and must be completed within 55 minutes. Test-takers analyse and interpret images, charts and text, make inferences based on the provided information and evaluate those inferences. At the end of the test, participants receive an individual performance report^(15)^. CT levels are calculated based on participants’ answers to the test questions. The overall CT level and scores for skills are measured on a 100-point scale^(16)^. A qualitative description of scores and scales is provided in Chart 1. Insight Assessment^(17)^ provides examples of questions. The HSRT has been used in several nursing studies^(7,13,18-21)^ as well as in research on other health care professions^(22-25)^. The HSRT shows content, construct and criterion validity^(15,26)^ and an internal consistency of 0.81^(13)^. Huhn, Black, Jensen, Deutsch^(26)^ found that the HSRT’s total score discriminated between expert and novice CT skills, confirming its construct validity.

**Chart 1:** The interpretation of Health Sciences Reasoning Test scores

50-62	63-69	70-78	79-85	86-100
**Not manifested**	**Weak**	**Moderate**	**Strong**	**Superior**
This result is consistent with possible insufficient test-taker effort, cognitive fatigue or possible reading or language comprehension issues.	This result is predictive of difficulties with educational and employment-related demands for reflective problem-solving and reflective decision-making.	This result indicates the potential for skills-related challenges when engaged in reflective problem-solving and reflective decision-making associated with learning or employee development.	This result is consistent with potential academic success and career development.	This result indicates CR skills that are superior to those of most test-takers. Skills at the superior level are consistent with the potential for more advanced learning and leadership.

**Chart 2:** Descriptions of critical thinking domains measured by the Health Sciences Reasoning Test

CT domain	Description	Nurse skill examples
Analysis	Analytical skills facilitate: identifying assumptions, reasons and claims; evaluating how they interact in the development of arguments; examining key elements in any situation and determining how they relate to one another; identifying and observing patterns and details; and gathering relevant information from speech, documents, signs, tables, charts, graphs and diagrams.	In clinical practice, nurses perform data analysis based on health indicators, signs and symptoms, including analysis of information on the health history and responses of patients under their care. Example: A nurse analyses a patient’s vital signs before deciding whether or not to administer a prescribed medication or requests an evaluation from the medical team.
Interpretation	Interpretive skills facilitate: comprehending and expressing the meaning of anything e.g., experiences, situations, communication (written messages, verbal and non-verbal exchanges), behaviours, data (graphs, diagrams, maps, charts, memes), events, judgements, conventions, beliefs, rules, procedures, criteria, and social interactions.	Nurses employ interpretation daily in all aspects of nursing, including the interpretation of signs and symptoms, health problems, test results and quantitative and qualitative data generated in the act of caring. Example: A nurse interprets a patient’s signs and symptoms to determine the priority nursing diagnoses to be considered for developing a care plan.
Inference	Inferential skills facilitate: elaborating conjectures and hypotheses; predicting the consequences of various options under consideration; identifying the logical consequences of assumptions; drawing conclusions from reasons, evidence, observations, experiences, values and beliefs using all forms of analogous, probabilistic, empirical and mathematical reasoning.	The inference skill is employed when nurses in clinical practice formulate hypotheses in the process of nursing diagnosis and when they observe changes in clinical picture and must infer what may be happening to patients. Example: A nurse decides what to do in a given situation in clinical practice by drawing conclusions based on professional experience. For instance, nurses may identify dysphagia in a patient with sequelae of stroke and suggest to the medical team that a nasogastric tube be inserted for feeding. Nurses infer the need for an intervention, which in this case is essential for the proper nutrition of patients.
Evaluation	Evaluative skills facilitate: assessing the credibility of claims that are made or published; assessing the quality of people’s reasoning when making arguments or giving explanations; evaluating the quality of many other important elements of a valid rationale, such as analysis, interpretation, explanation, inference, options, opinions, beliefs, assumptions, proposals and decisions.	The evaluation skill, like the others, is of fundamental importance in the clinical practice of nursing and is considered a step in the nursing process and a methodological tool that organises and guides nurses’ work. Assessment is employed in all a nurses’ actions to verify patients’ conditions and responses to interventions in the care plan as well as the results achieved. Example: A nurse in clinical practice assesses a patient’s responses to a specific implemented care plan to identify its progress and the need to include or exclude interventions to achieve better results.
Explanation	Explanatory skills facilitate: justifying what one chooses to do or believe by presenting convincing arguments guided by evidence, concepts, methodology, criteria, contextualisation, values, reasons and assumptions.	The explanation skill is frequently used by nurses in clinical practice to justify providing specific care and in implementing specific interventions. Through explanation, nurses guide their teams and patients towards safe and quality care. Example: A nurse must educate a team on the need for special care for a patient and must explain the rationale and purpose of implementing such care to obtain better results and speed up patient recovery.
Inductive Reasoning	Inductive reasoning skills facilitate: decision-making in contexts of uncertainty. Inductive decisions may be grounded on analogies, case studies, prior experience, statistical analysis, simulations, hypotheses, reliable testimony, experiences, events, recognisable patterns in a set of events, experiences, symptoms and behaviours. Conclusions guided by inductive reasoning may be mistaken, but, although it does not provide certainty, it can provide a solid basis for confidence in conclusions about people and a reasonable basis for action.	Inductive reasoning based on professional experience informs a nurses’ decision-making when they must take the best possible decision in solving a problem. Example: A nurse evaluates a patient and, making a correlation with previous experiences, takes the best decision to solve the problem presented by patients.
Deductive Reasoning	Deductive reasoning skills facilitate: decision-making in precisely defined contexts in which rules, operating conditions, core beliefs, values, policies, principles, procedures and terminology completely determine the outcome. Deductive validity describes a conclusion that absolutely cannot be false if the initial assumptions or premises are true. Deductive validity leaves no room for uncertainty unless we change the very meaning of our words or the grammar of our language.	Deductive reasoning is grounded on using facts in evaluating and solving problems. In the application of deductive reasoning, nurses take precise decisions to solve problems. Example: A nurse assesses the problem presented by a patient based on facts and scientific criteria for decision-making in clinical practice.
Numeracy	Numeracy skills facilitate: making judgements based on quantitative information in several contexts; applying knowledge based on numbers, arithmetic, measures and mathematical techniques in situations that require the interpretation or evaluation of information; understanding how quantitative information is gathered, manipulated and represented visually (as in graphs, charts, tables and diagrams).	Quantitative reasoning is frequently employed by nurses in clinical practice when evaluating quantitative data, performing drug dose calculations and determining the number of staff in a clinical unit to provide safe, quality care to patients. Examples: A nurse in collaboration with a nursing team calculates the dose of a drug to be administered and consults the guidelines before administration. A nurse determines an adequate number of professionals for a team that will provide nursing care in an Intensive Care Unit.

### Completed Health Sciences Reasoning Test validity

Following the HSRT guidelines, tests with less than 60% of the items completed or with a completion time of less than 15 minutes were considered invalid, as they do not provide reliable assessment of participants’ clinical reasoning^(15)^.

### Data collection

Participants completed the sociodemographic data via Google Forms and received usernames and passwords to access and complete the HSRT. Each participant was given a detailed score report upon completing the assessment. Insight Assessment^(27)^ provides a sample report.

Sociodemographic data were collected for age, sex, colour/race, professional course, course level (undergraduate or graduate study), semester of study, public or private educational institution and employment status. Moreover, participants were asked whether they had previously participated in courses to develop CT skills and whether they believed it was important to develop CT skills in health sciences education and professions.

### Data analysis and statistics

The statistical analysis of participants’ responses was conducted by Insight Assessment. The researchers received a report of the results, which were then manually transferred to IBM SPSS version 21.0 (SPSS Inc., Chicago, IL, USA) for further analysis. Descriptive statistics were obtained for sociodemographic data and CT domains. The Kolmogorov-Smirnov test verified the normality of the outcome variables (CT domains). Continuous variables were described by mean and standard deviation, and categorical variables, by absolute and relative frequency. Student’s t-test was used to compare the means. The correlation between variables was assessed using the Pearson correlation coefficient (PCC). The adopted significance level was 5% (p ≤.05)^(28-29)^.

### Sample size calculation

The sample size calculation was performed using the WinPEPI (Programs for Epidemiology for Windows) version 11.43, based on a study by Hanlon et al.^(30)^. At least 88 students were needed considering a significance level of 5%, standard deviation estimated at 4 points for the total HSRT score, margin of error of 5%, power of 80% and a minimum effect size of 0.6, and standard deviation.

## RESULTS

### Participant sociodemographic profile

Of the 160 participants who completed the HSRT, 23 were excluded because they did not provide an eligible test (answered less than 60% of the items or completed the test in less than 15 minutes). Of the 137 participants that provided an eligible test 48 did not meet the inclusion criteria (UNS). The final study sample comprised 89 UNS (66 female) with a mean age of 26.6 years. Sixty-four attended public educational institutions. All participants considered CT an important skill to be developed in nursing education.

### Health Sciences Reasoning Test domains

Overall, participants’ CT skills scores were moderate (Table 2). Induction had the highest mean score (76.4), followed by Explanation (74.2) and Inference (71.3), all with moderate level mean scores. The CT domains with the lowest mean score was Numeracy (63.1), followed by Deduction (64.6), Interpretation (66.0), Evaluation (67.8) and Analysis (69.1).

**Table 1:** Sociodemographic data

Variable	n = 89
Age (years) - mean (standard deviation)	26.6 (7.9)
Sex - n (%)	
Male	23 (25.8)
Female	66 (74.2)
Ethnicity - n (%)	
White	65 (73.0)
Brown	19 (21.3)
Black	5 (5.6)
Education period^*^ - n (%)	
Initial (1^st^ to 3^rd^ semester)	28 (31.5)
Intermediate (4^th^ to 7^th^ semester)	33 (37.1)
Final (8^th^ to 10^th^ semester)	28 (31.5)
High school education - n (%)	
Public	64 (71.9)
Private	25 (28.1)
Employed - n (%)	
No	42 (47.2)
Yes	47 (52.8)
Performed course in critical thinking - n (%)	
No	81 (91.0)
Yes	8 (9.0)
Considers critical thinking an important competence to be developed in nursing education - n (%)	
No	0 (0.0)
Yes	89 (100)
Time of test performance (minutes) - mean (standard deviation)	50.6 (8.4)

**Table 2:** Overall results of the Health Sciences Reasoning Test

Domain	Mean ± SD	Superior	Strong	Moderate	Weak	Not manifested
		n (%)	n (%)	n (%)	n (%)	n (%)
Analysis	69.1 ± 8.3	3 (3.4)	5 (5.6)	34 (38.2)	31 (34.8)	16 (18.0)
Interpretation	66.0 ± 6.6	0 (0.0)	1 (1.1)	22 (24.7)	33 (37.1)	33 (37.1)
Inference	71.3 ± 6.5	3 (3.4)	5 (5.6)	35 (39.3)	42 (47.2)	4 (4.5)
Evaluation	67.8 ± 6.3	0 (0.0)	1 (1.1)	31 (34.8)	35 (39.3)	22 (24.7)
Explanation	74.2 ± 9.2	13 (14.6)	13 (14.6)	33 (37.1)	21 (23.6)	9 (10.1)
Induction	76.4 ± 7.3	8 (9.0)	26 (29.2)	39 (43.8)	14 (15.7)	2 (2.2)
Deduction	64.6 ± 6.8	0 (0.0)	2 (2.2)	17 (19.1)	40 (44.9)	30 (33.7)
Numeracy	63.1 ± 6.3	0 (0.0)	2 (2.2)	12 (13.5)	46 (51.7)	29 (32.6)
Overall	70.7 ± 5.7	0 (0.0)	13 (14.6)	36 (40.4)	35 (39.3)	5 (5.6)

### Correlation results

In the correlation analyses between UNS’ CT domains and their age, employment, course period, time of testing performance, sex, level of education and previous course in CT, only the analyses between UNS’ CT and their course period yielded a statistically significant correlation (*p* = .001) (Table 3).

**Table 3:** Correlation between Health Sciences Reasoning Test and course period

HSRT domain	Course period	
	PCC^*^	*p* value
Analysis	.320	.002
Interpretation	.095	.375
Inference	.283	.007
Evaluation	.179	.094
Explanation	.154	.149
Induction	.373	<.001
Deduction	.193	.070
Numeracy	.209	.049
Overall	.400	<.001

* *Pearson correlation coefficient.*

Table 3 shows a statistically significant positive correlation between the course period and the HSRT Induction domain and overall scores. The further the UNS were in the course period (towards the end of the course), the higher the scores they achieved.

## DISCUSSION

This study used the HSRT to examine Brazilian UNS’ CT levels. The results show that participants demonstrated an overall moderate level of CT skills, but the level in 5 of the 8 domains was weak. Our results also show a positive correlation between the HSRT overall scores and the Induction domain and the course period in nursing education.

Regarding the overall CT levels, our results align with those of previous studies^(13,19-22,31)^, but few studies^(13,22)^ have examined in detail the distribution of CT levels between participants and in the various HSRT domains. Most of our participants (55%) had a moderate to strong level of CT based on the HSRT results. Still, 45% of participants did not achieve the minimum expected CT level for UNS. Our results indicate that Brazilian UNS performed worse on the HSRT than UNS from other countries, when compared to other studies^(7,13,19)^. A study with 134 participants in Australia found that 9.7% the participants had a weak CT level^(13)^, while, in our study, five times as many participants manifested a weak CT level. The reason why Brazilian UNS achieve so low score in CT compared to students from other countries should be investigated.

Our results indicate that Brazilian UNS may have a high probability of failing in their study progression or dropping out of nursing education due to low CT level. Several studies have found a correlation between CT level and academic capability, clinical performance and progression^(13,32-34)^. Pitt, Powis, Levett-Jones, Hunter^(13)^ and Jones, Morris^(35)^ found that CT levels could predict UNS drop-out or retention. A mandatory CT test at admission, during and at completion of the nursing programme, may reduce the time and resources educators expend on UNS who are unlikely to complete the education. Alternatively, the results of a mandatory CT test upon admission to the nursing programme could be used to help those with greater difficulties develop the minimum required CT score by individually tailoring teaching strategies.

In our study, only 14.6% of participants achieved a strong score on the HSRT test and none achieved the superior level, while a study with 152 participants in the USA found that 29% of participants achieved superior CT level^(19)^. According to the definition of CT levels (Chart 1), our results indicate that few participants had the necessary skills to pursue an academic career. The developers of the HSRT claim that those who achieve a strong or superior score on the CT test have the potential to engage in an academic career, but several studies, including ours, show that few participants who take the CT test achieve those levels. These results can negatively impact the number of professionals able to pursue an academic carrier and may be one explanation for the increasing shortage of academic professionals in nursing education^(36-37)^. Fang and Kesten^(37)^ predict a huge impact of retiring educators on the faculty workforce in the next few years and suggest that the younger educators who are likely to replace them may lack doctoral degrees, professor competence and have less ability for graduate level teaching. This underlines the importance of nurturing CT skills in UNS.

Thirty-seven percent of our participants did not manifest interpretation skills in the HSRT test; their scores ranged from weak (37.1%) to strong (1.1%); and none of the participants achieved a superior score. Employing interpretation in combination with the other CT skills is essential for professional activities in health care. Strategies to enhance UNS’ CT skills must be discussed and quickly adopted. To train UNS in CT, educators must be competent, possess in-depth knowledge of CT skills and adopt pedagogical strategies for teaching those skills effectively. Several studies suggest that teaching approaches such as activities based on evidence-based nursing^(38)^, PBL^(39)^, simulation^(20)^, metacognition^(40)^ and team-based learning^(22)^ can increase overall CT skills. Furthermore, to develop teaching strategies that support UNS in developing CT, it is vital to recognise areas for improvement in current nursing education^(11).^

Our results yielded predominantly moderate and strong scores in the ability to explain, so the UNS in the sample clearly had good skill levels in activities and decision-making that require the practical application of explaining thoughts and resolving problems. In line with previous studies^(7,13,20,22,31)^, we found that the explanation skill appears to be well developed and that our participants achieved the highest score on the induction skill.

In the present study, UNS had the lowest CT scores in Deduction and Numeracy; none achieved a superior score for those skills and few were rated strong. These results partly align with those of other studies in which UNS scores in deduction skills were weak^(7,13,20)^ or not manifested^(31)^. CT skills are essential for UNS’ performance in clinical practice, and studies indicate a weakness in the training process in relation to the development of these skills. Our scores indicate that UNS may have a poor ability to perform drug calculations and quantitative analysis, which can greatly compromise patient safety and quality of care^(41-42)^. Therefore, it is essential to encourage UNS’ development in quantitative reasoning through active teaching-learning strategies^(20,22,38-40)^ and through focused activities that include quantitative reasoning and calculation^(41-42)^. Such steps can reduce future problems and the negative consequences of poor nursing resulting from inadequate numeracy.

We found a positive correlation between HSRT overall scores, the Induction domain and the length of nursing education period. Although our sample was small, these results are in line with prior studies with more participants, confirming these correlations^(7,13,35)^. These results suggest that UNS increase their CT levels throughout their nursing education. However, educational institutions must ensure that, when UNS complete their education, they are able to provide safe nursing care to their patients, and a high level of CT skills may correlate with patient safety^(43-44)^.

We assume that the most motivated UNS that may have believed that they were good critical thinkers (those who were curious to learn their CT level) were interested in participating in our research, therefore, our sample may be biased. When motivated students do not manage to complete the HSRT (14.5%), we must be concerned about their level of knowledge and competence when they finish their college education and must consider how education influences CT. Their poor performance may be explained by stress related to time pressure when taking the test, but this phenomenon would also be expected in testing an entire UNS population. CT tests as a mandatory part of the nursing education curriculum could be important to gain knowlegde of CT levels in a normally distributed (non-biased) UNS population.

### Study limitations

A limitation of our study was the small number of participants. It was challenging to recruit participants, due to the COVID-19 pandemic, which prevented us from holding personal meetings to explain the study and motivate UNS to participate. Reminders and new advertisements were generated in order to increase the participant numbers. Another recruitment challenge may be related to time required and difficulty of completing the HSRT. In this pilot study, more than 14% of participants were not able to complete the HSRT, and, therefore, their data were excluded. Convenience sampling was used, which may have produced an over- or underrepresenting sampling bias of students with distinct CT profiles.

Another limitation is the inclusion UNS from different universities (public and private) and different study programmes, as it may reduce the study reliability. When a statistically significant positive correlation between CT and education period is confirmed, we can assume that Brazilian UNS may have even lower CT skill levels than indicated by this study results and compared to similar studies with UNS target groups. Our results cannot be generalised due to sample size, target group and bias resulting from recruiting conditions.

### Contributions to nursing

Due to the importance of CT skills to the nursing profession, it is important to map UNS’ CT levels and improve teaching strategies when needed.

## CONCLUSIONS

It is important to encourage and ensure the development of CT in UNS because of global changes in health care and nursing practice, use of more advanced technology and an increase in patient acuity^(45)^. This is the first study using the HSRT to investigate CT levels in Brazilian UNS. Our study showed that Brazilian UNS have unsatisfactory CT levels that need to be improved in the most domains measured by the HSRT. Further studies should investigate the reasons why Brazilian UNS achieve low CT scores, to enable educators to tailor teaching strategies to increase CT levels. Although the correlation between CT and course period is positive, further studies is needed to investigate whether Brazilian UNS have achieved the satisfactory level in all CT domains at the end of the nursing programme. Furthermore, a routine for measuring CT in educational institutions should be implemented. However, further study is necessary to confirm our results. Strategies to ensure CT encouragement in nursing education may crucially contribute to the completion of nursing education by UNS, to recruitment, to success in academic careers and to patient safety.
